# MEK1 signaling promotes self-renewal and tumorigenicity of liver cancer stem cells via maintaining SIRT1 protein stabilization

**DOI:** 10.18632/oncotarget.7972

**Published:** 2016-03-07

**Authors:** Jiamin Cheng, Chungang Liu, Limei Liu, Xuejiao Chen, Juanjuan Shan, Junjie Shen, Wei Zhu, Cheng Qian

**Affiliations:** ^1^ Institute of Pathology and Southwest Cancer Center, Southwest Hospital, Third Military Medical University, Chongqing, 400038, China

**Keywords:** hepatocellular carcinoma (HCC), cancer stem cells (CSCs), MEK1 signaling, SIRT1, proteasome degradation

## Abstract

Hepatocellular carcinoma (HCC) is the third leading cause of cancer death. This high mortality has been commonly attributed to the presence of residual cancer stem cells (CSCs). Meanwhile, MEK1 signaling is regarded as a key molecular in HCC maintenance and development. However, nobody has figured out the particular mechanisms that how MEK1 signaling regulates liver CSCs self-renewal. In this study, we show that inhibition or depletion of MEK1 can significantly decrease liver CSCs self-renewal and tumor growth both *in vitro* and *vivo* conditions. Furthermore, we demonstrate that MEK1 signaling promotes liver CSCs self-renewal and tumorigenicity by maintaining SIRT1 level. Mechanistically, MEK1 signaling keeps SIRT1 protein stabilization through activating SIRT1 ubiquitination, which inhibits proteasomal degradation. Clinical analysis shows that patients co-expression of MEK1 and SIRT1 are associated with poor survival. Our finding indicates that MEK1-SIRT1 can act as a novel diagnostic biomarker and inhibition of MEK1 may be a viable therapeutic option for targeting liver CSCs treatment.

## INTRODUCTION

Hepatocellular carcinoma (HCC) is not only the fifth most familiar malignant tumor across the world but also the third cause of cancer-related death in Asia, especially in China [1, 2]. Although specific surgery has been established as the first-line treatment for HCC, HCC patients still suffer from the high morbidity of postoperative recurrence and therapy resistance which both lead to an indefinite survival time [3–6]. Recently, cancer stem cells (CSCs) or tumor initiating cells (TICs), which are defined by their tumor-initiating feature, are responsible for driving the tumor growth, therapy resistance, recurrence, metastasis, and causing poor patients outcome [7]. Our previous study demonstrated that transcription factor Nanog was a liver CSCs marker which regulated self-renewal *in vitro* and *vivo* [8]. Therefore, it may be crucial to identify cell-state-specific features that may render CSCs susceptible for selectively therapeutic intervention.

Mitogen-activated protein kinase 1 (MAPK1/MEK1) belongs to the MAPKs family. They are dual specificity enzymes that phosphorylate threonine and tyrosine residues within the activation loop of their MAP kinase substrates [9]. Dysregulation of MEK1 has been implicated in many diseases, including cancer. MEK1 is up-regulated in a number of tumors' genesis. It is regarded as an oncogene which promotes cancer formation, progression and therapy resistance. MEK1-YAP interaction is critical for HCC proliferation and tumorigenesis [10]. Activation of MEK1/ERK signaling promotes transforming growth factor Beta 1-modulated growth, collagen turnover, and differentiation of stem cells from Apical Papilla of human tooth [11]. Consistently, MEK1 is also reported to be associated with mesenchymal stem cells proliferation, collagen synthesis and spermatogonia stem cells self-renewal [12–14]. Scientists prove that knockdown Calcium channel α2δ1 subunit reduces HCC CSCs sphere formation and tumorigenicity upon MAPK pathway [15]. However, the underlying mechanism of MEK1 functions in liver CSCs is still elusive.

The sirtuin (s) is a highly conserved family of NAD-dependent enzymes. It contains seven family members (SIRT1-7), which control various cellular processes including cell cycle, cellular metabolism, cell proliferation, differentiation, genome stability and cancer [16–18]. Recently, more and more researches demonstrate that sirtuin (s) plays an important role in maintaining stem cells or differentiation statement, while little is known about how MEK1 influences liver CSCs self-renewal. In this study, we investigate the functional contribution of MEK1 in human liver CSCs self-renewal and propagation. We uncover an MEK1-mediated SIRT1 protein stabilization underlying CSC state which can be associated with liver CSCs maintenance and poor patients' prognosis.

## RESULTS

### MEK1 inhibition reduces proliferation and self-renewal of liver CSCs

**Figure 1:**
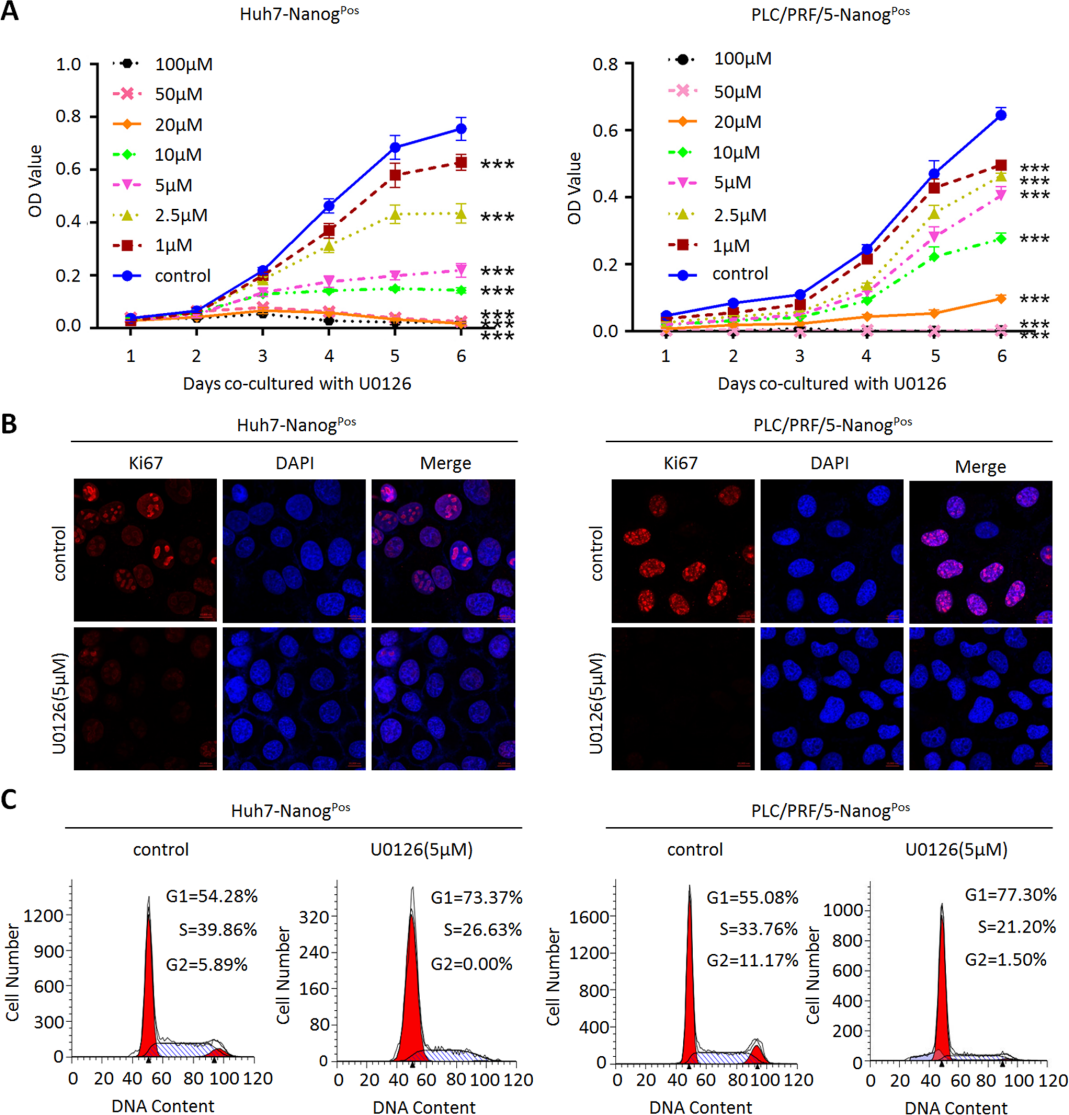
MEK1 inhibitor decreases liver CSCs proliferation ability *in vitro* (**A**) Huh7-Nanog^Pos^ and PLC/PRF/5-Nanog^Pos^ cells under different U0126 concentrations treatment as indicated (0 μM, 1 μM, 2.5 μM, 5 μM, 10 μM, 20 μM, 50 μM, 100 μM) were seeded (1 × 10^3^) and cultured for another 6 days before analyzed with CCK8. (**B**) Huh7-Nanog^Pos^ and PLC/PRF/5-Nanog^Pos^ cells were cultured with or without 5 μM U0126 for 48 hours. Cells were harvested for immunofluorescence (IF) analysis by anti-Ki67 antibodies. Scale bar, 10 μm. (**C**) Cell cycle profiles of 5 μM U0126 treated or DMSO treated (negative control) Huh7- and PLC/PRF/5-Nanog^Pos^ cells followed by treatment with Sodium butyrate. Percentage in each histogram indicates the portion of cells remaining in each cell cycle phase.

In order to test whether the altered MEK1 activity regulates proliferation and self-renewal of liver CSCs, we tested the specific MEK1 inhibitor-U0126 on the liver CSCs (Nanog^Pos^) which were isolated by our previously constructed P_Nanog_-GFP lentivirus reporter system [8]. Our results showed that U0126 could inhibit proliferation of liver CSCs in dose-dependent manner (Figure 1A). Meanwhile, Ki-67 was dramatically reduced in liver CSCs after treatment with U0126 (Figure 1B). Cell cycle analysis showed that U0126 treatment caused a significant reduction of S and G2/M phases and increase of G0/G1 phase (Figure 1C). These data suggested that the inhibition of proliferation in liver CSCs by U0126, at least partly, was due to interference with the cell cycle.

**Figure 2:**
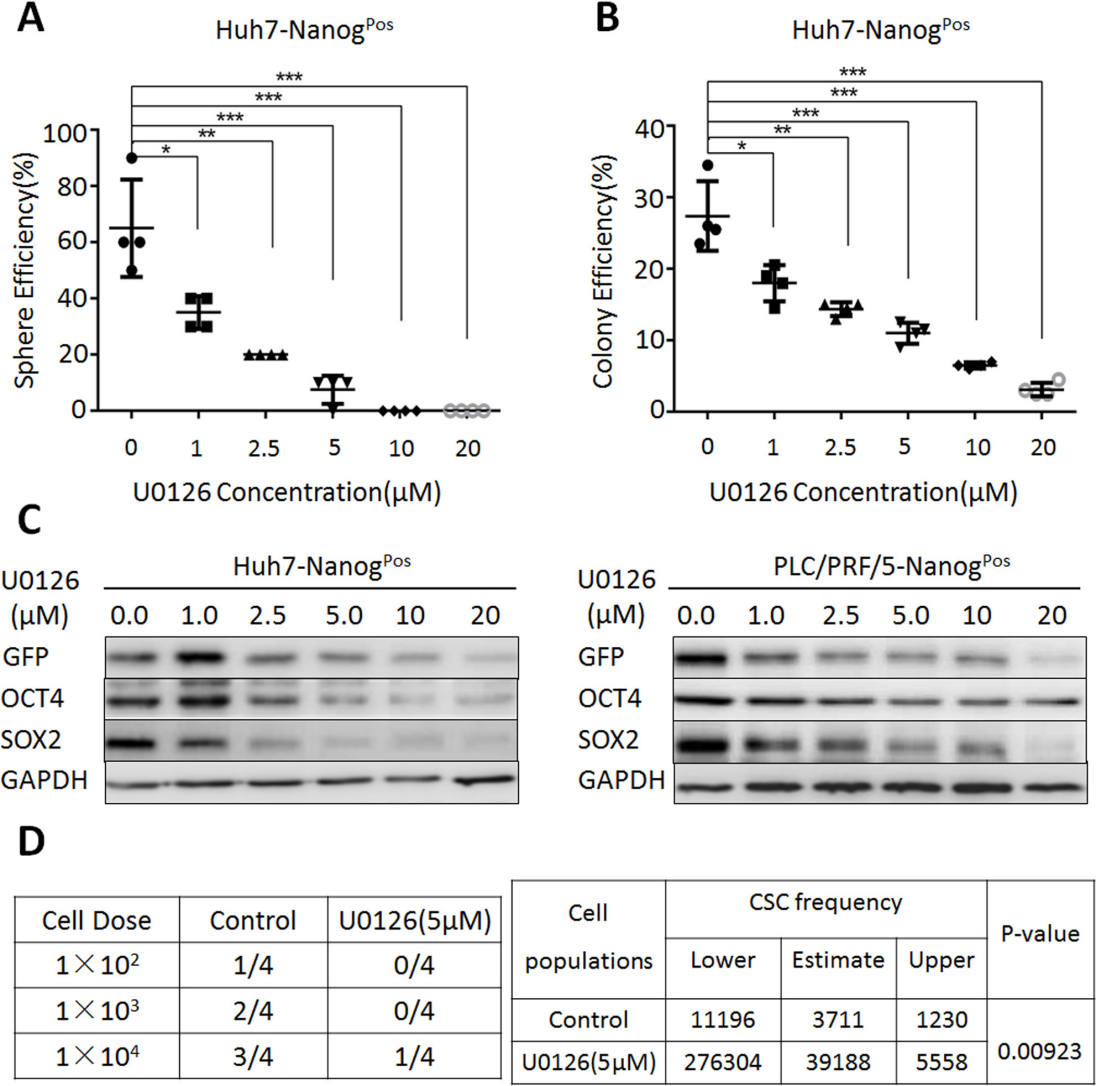
MEK1 signaling activity is required for the maintenance of liver CSC self-renewal (**A**) Huh7-Nanog^Pos^ cells were co-cultured with various concentrations of U0126 (0 μM, 1 μM, 2.5 μM, 5 μM, 10 μM, 20 μM) in sphere-forming conditions for 7 days, counted at the same magnification. (**B**) Huh7-Nanog^Pos^ cells were treated with different concentrations of U0126 (0 μM, 1 μM, 2.5 μM, 5 μM, 10 μM, 20 μM) and grown for 14 days. Cells were stained with crystal violet and counted. (**C**) Western blot analysis of stemness protein expression in Huh7- and PLC/PRF/5-Nanog^Pos^ cells, which co-cultured with various concentrations of U0126 (0 μM, 1 μM, 2.5 μM, 5 μM, 10 μM, 20 μM) for 48 hours. (**D**) Huh7-Nanog^Pos^ cells were treated with 5 μM U0126 for 14 days, while the negative control treated with DMSO for 14 days. Then we subcutaneous injected 1 × 10^2^, 1 × 10^3^, 1 × 10^4^ cells into NOD-SCID mice. After 30 days, we harvested and counted the tumors. Extreme Limiting Dilution Analysis was acquired from http://bioinf.wehi.edu.au/software/elda/.

Furthermore, we examined the effect of MEK1 inhibition on the self-renewal ability in liver CSCs population. Our results demonstrated that U0126 dramatically decreased sphere formation efficiency (Figure 2A) and clone formation efficiency (Figure 2B), when the concentrations of U0126 from 1 μm to 20 μm were used. Cell survival analysis showed that these doses didn't significantly decrease liver CSCs proliferation (Figure S1). Contrary to liver CSCs, we found that low concentration of U0126 treatment did not reduce clone and sphere formation efficiency on liver non-CSCs (Figure S2). In addition, we found that the protein levels of stemness genes (OCT4, SOX2, GFP) were markedly decreased, when liver CSCs treated with different concentrations of U0126 (Figure 2C). To confirm these results, we used another MEK1 inhibitor-PD98059. Our data showed that PD98059 could also lead the significant reduction of sphere formation efficiency, clone formation efficiency and stemness genes expression (Figure S3). We also found that both MEK1 inhibitors decreased phospho-MEK1 level, but not MEK1 level (Figure S4). To further examine U0126 could induce similar effects *in vivo*, we chosen HCC cell line-Huh7 in NOD/SCID mice of xenograft model. Compared to the control, treatment of liver CSCs with U0126 could significantly tumor growth (Figure 5G and 5H). Limiting dilution analysis showed that CSC frequency was significantly reduced in liver CSCs after treatment with U0126 (Figure 2D). These data indicated that for liver CSCs populations, the ability to active MEK1 signaling activity was a critical determinant of their proliferation and tumor-initiating potential.

### Participation of MEK1 in the self-renewal of liver CSCs

**Figure 3:**
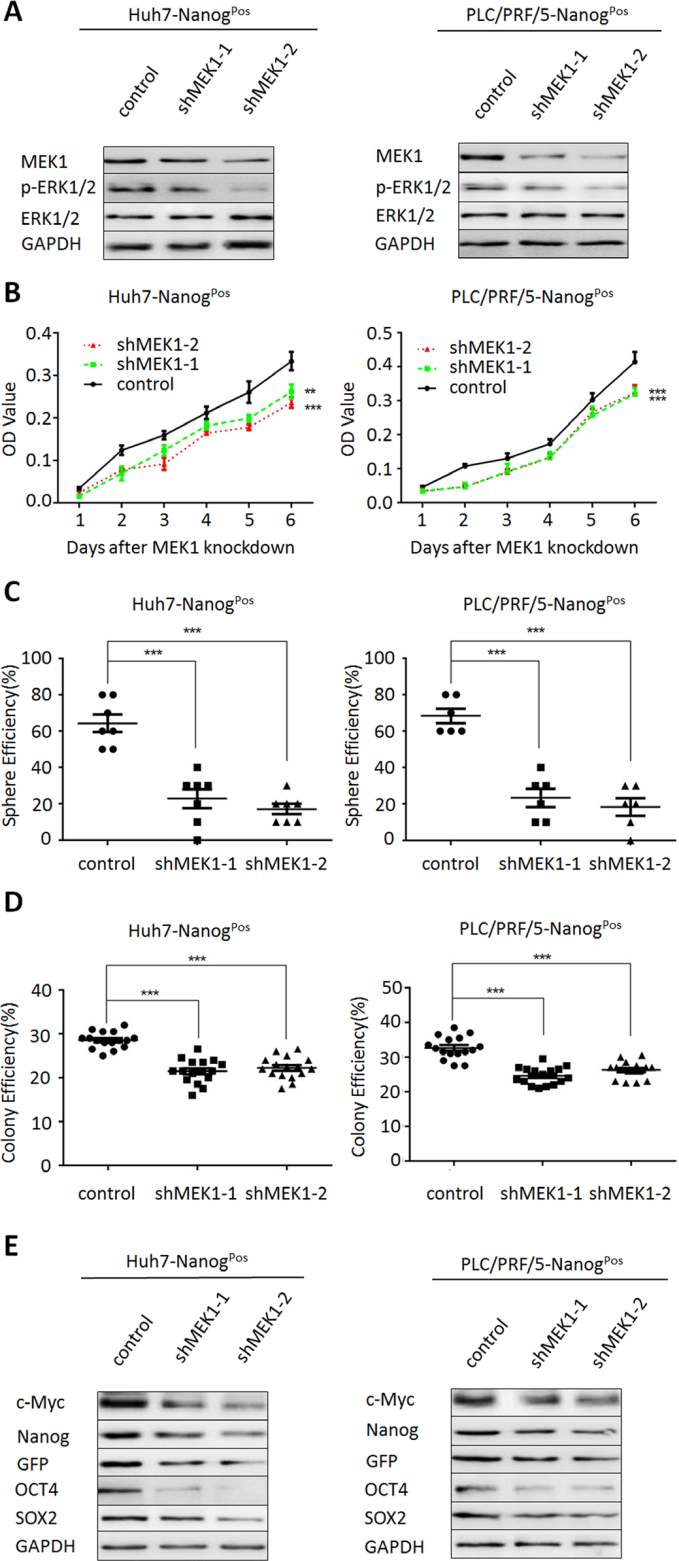
MEK1 knockdown suppresses liver CSC self-renewal and tumorgenetic capacity (**A**) Western blot analysis MEK1 and the substrate ERK1/2 expression in Huh7- and PLC/PRF/5-Nanog^Pos^ cells which depleted MEK1 with two individual lentiviruses for 48 hours. (**B**) Effect of MEK1 knockdown on cellular growth rates of Huh7- and PLC/PRF/5- Nanog^Pos^ cells. CCK8 assay was performed after transfection with indicated times. Cell lysates were obtained from cells transiently transfected with either MEK1 shRNA or negative control shRNA. (**C**) Huh7- and PLC/PRF/5- Nanog^Pos^ cells which transfected with MEK1 shRNA or negative control shRNA cultured under non-adhesive culture system for 7 days. (**D**) Huh7- and PLC/PRF/5-Nanog^Pos^ cells were transduced with lentiviruses expressing the indicated shRNA. Cells were grown for 14 days and stained with crystal violet. (**E**) Western blot analysis of stemness-related proteins in MEK1-depleted cells, relative to control.

To further test whether the MEK1 signaling was important to maintain self-renewal and tumor growth of liver CSCs, we silenced MEK1 expression with two shRNA (shMEK1-1 and shMEK1-2) on liver CSCs which were isolated from Huh-7 and PLC/PRF/5. Results showed that MEK1 and phospho-ERK1/2 level was significant decreased when knockdown of MEK1 expression in liver CSCs (Figure 3A), while ERK1/2 expression remained the same, and cell proliferation was also inhibited compared with control (Figure 3B). Moreover, knockdown of MEK1 expression in the liver CSCs suppressed both sphere formation (Figure 3C) and clone formation (Figure 3D). In addition, knockdown of MEK1 expression in liver CSCs resulted in the decreasing expression of stem cells markers, including Nanog, OCT4, c-Myc and SOX2 (Figure 3E). Overall, these data further showed that MEK1 depletion played a negative role in the self-renewal of liver CSCs.

### MEK1 promotes liver CSCs self-renewal relaying on histone deacetylase SIRT1

**Figure 4:**
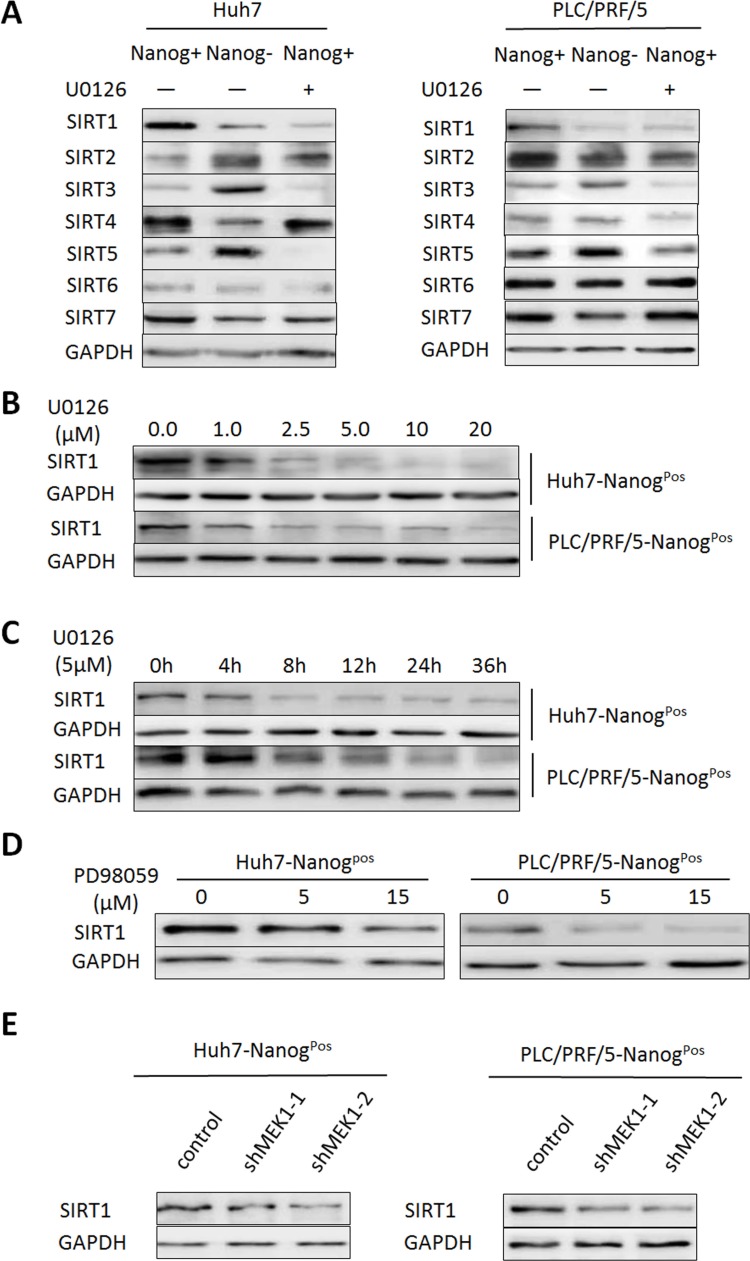
MEK1 mainly promotes SIRT1 expression in HCC population (**A**) Western blot analysis the sirtuins expression level in CSCs and non-CSCs, compared with U0126 inhibited CSCs. (**B**) Western blot analysis SIRT1 expression in CSCs after co-cultured with indicated concentration of U0126 or PD98059 (**D**) for 48 hours. (**C**) Western blot analysis SIRT1 expression in CSCs which cultured with 5 μM U0126 for indicated times. (**E**) Western blot analysis SIRT1 expression in CSCs transduced with lentiviruses expressing the indicated shRNA for 48 hours. Those experiments were repeated in two HCC cell lines (Huh7 and PLC/PRF/5).

Recently, more and more evidences suggest that sirtuins family plays a key role in CSCs self-renewal, tumor progression and poor outcome in HCC [19]. To explore whether sirtuin (s) was involved in self-renewal and tumorigenesis of liver CSCs which mediated by MEK1, we examined expression diversity of SIRTs in CSCs, non-CSCs or U0126 treated CSCs. As shown in Figure 4A, SIRT1, 2, 3, 4, 5 and 7 were differentially expressed in CSCs and non-CSCs cells. SIRT1/5 was downregulated in liver CSCs after U0126 treatment. However, we found that SIRT5 was lower expressed in CSCs cells than non-CSCs cells (Figure 4A). Considering SIRT1 activity was essential for maintaining growth and self-renewal of liver CSCs, so we focused on analyzing whether SIRT1 was involved in promoting liver CSCs self-renewal and tumor development mediated by MEK1. Our results indicated that treatment of liver CSCs with U0126 decreased SIRT1 protein level at a dose or time-dependent manner (Figure 4B and 4C), but not SIRT1 mRNA level (data not shown). In addition, we also found that treatment with PD98059 or knockdown of MEK1 expression reduced SIRT1 protein level (Figure 4D and 4E).

**Figure 5:**
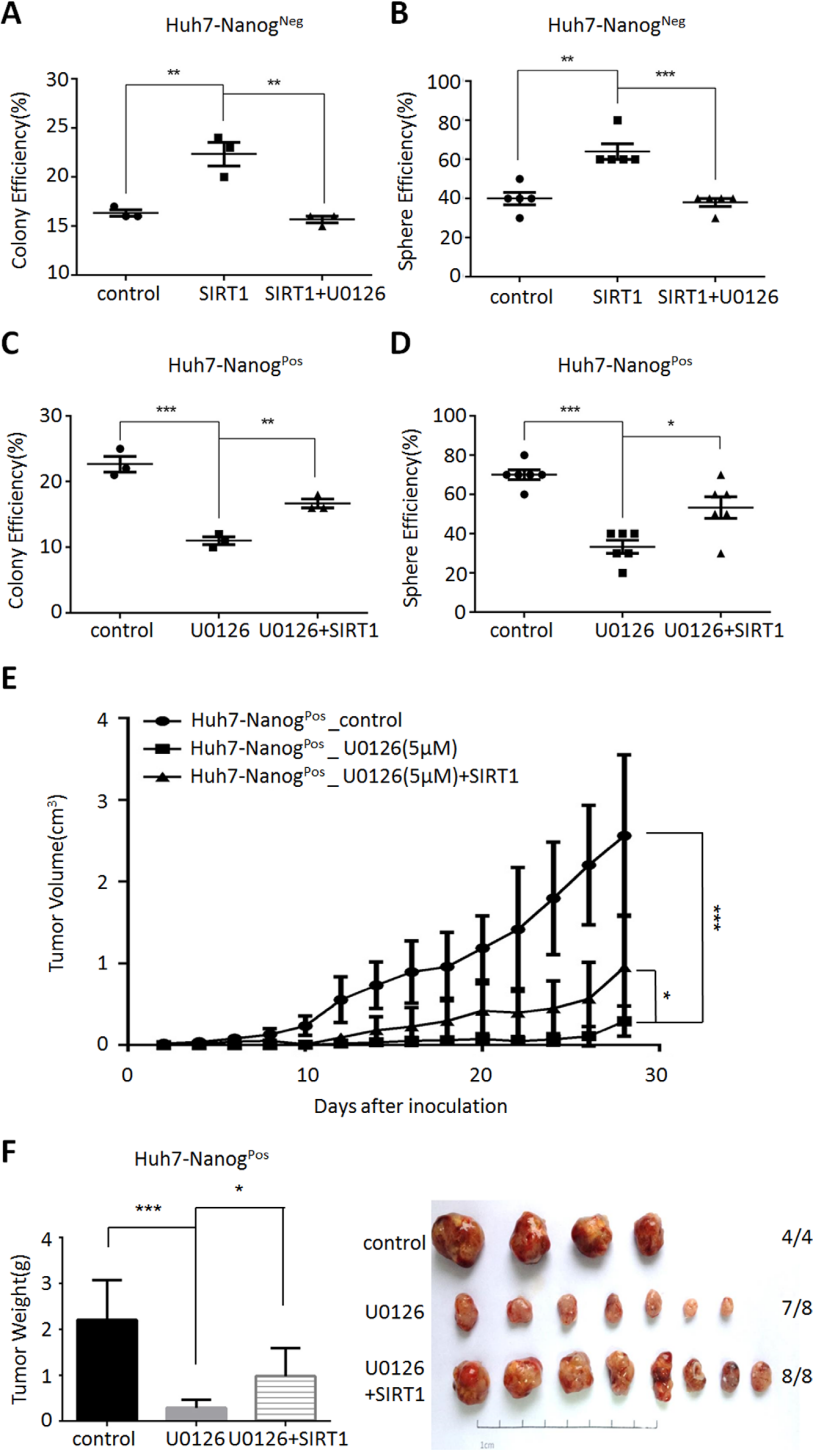
MEK1 maintains liver CSC self-renewal dependent on SIRT1 Non-CSCs (Huh7-Nanog^Neg^ cells) were prepared with overexpression SIRT1, and grown with 5 μM U0126 or DMSO for 48 hours. Liver CSCs (Huh7-Nanog^Pos^ cells) co-cultured with 5 μM U0126 for 24 hours previously and overexpression SIRT1 for next 24 hours. Colony analysis of CSCs (**C**) and non-CSCs (**A**) which were cultured for 14 days and stained with crystal violet. Sphere analysis of CSCs (**D**) and non-CSCs (**B**) which were cultured for 7 days in non-adhesive culture system. All counting were performed in triplicate. (**E**–**F**) CSCs, MEK1 inhibition CSCs and SIRT1 overexpression while MEK1 inhibition CSCs were prepared for 14 days, then subcutaneous injected in NOD-SCID mice (CSCs control group 4 mice, other two groups 8 mice each). Tumor sizes were measured with calipers in three dimensions every other day. Tumor volumes were calculated using the formula: tumor volume (cm^3^) = 0.52 × (W) ^2^ × (L), where L is length and W is width. We counted and weight the tumors, 30 days later.

Next, we investigated the functional impact of SIRT1 in MEK1-induced self-renewal and tumor initiating ability. Firstly, we examined whether SIRT1 activation was critical for those effects in liver non-CSCs cells. The results revealed that SIRT1 overexpression increased clone and sphere formation efficiency (Figure 5A and 5B), but these effects were reversed by the function of MEK1 inhibitor-U0126 (Figure 5A and 5B). In addition, we also proved that MEK1 inhibition down-regulated the efficiency of colony or sphere in CSCs, which was then rescued by reconstituted expression of SIRT1 (Figure 5C and 5D). These similar results were also found *in vivo* condition (Figure 5E and 5F). These data indicated that MEK1 maintained liver CSCs self-renewal and tumorigenesis through SIRT1.

### MEK1 enhances SIRT1 stability

**Figure 6:**
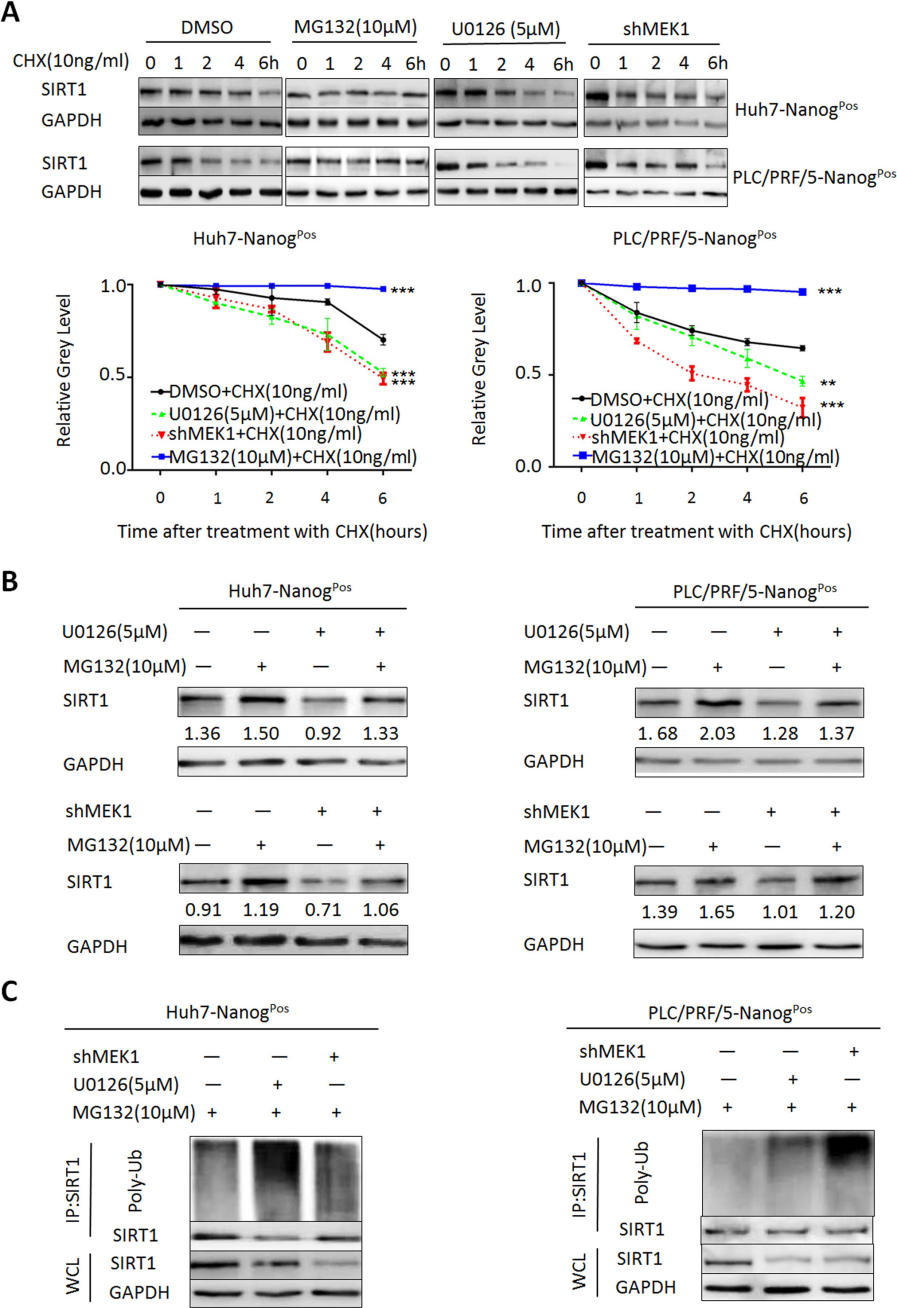
MEK1 keeps SIRT1 protein stability through proteasomal degradation inhibitory (**A**) We co-cultured proteasome inhibition, MEK1 inhibition or knockdown liver CSCs with CHX (10 ng/ml) for indicated times. Western blots analyzed expression of SIRT1. Grey level was measured triplicated independently. (**B**) Analysis of SIRT1 expression in liver CSCs by western blots. MEK1 deletion or inhibition (U0126, 5 μM) CSCs was cultured for 48 hours, then combined with or without Proteasome inhibitor (MG132, 10 μM) for 8 hours, before harvested. Those proteins were compared with CSCs of DMSO treatment with or without MG132. Grey level was measured and marked. (**C**) MEK1 inhibition or knockdown CSCs (Huh7- and PLC/PRF/5-Nanog^Pos^ cells) were treated with 10 μM MG132 for 8 hours before harvest. Total protein extracts from an equivalent number of seedlings were prepared for Co-IP in same conditions and analyzed using immunoblot with the Poly-Ub antibody.

**Figure 7:**
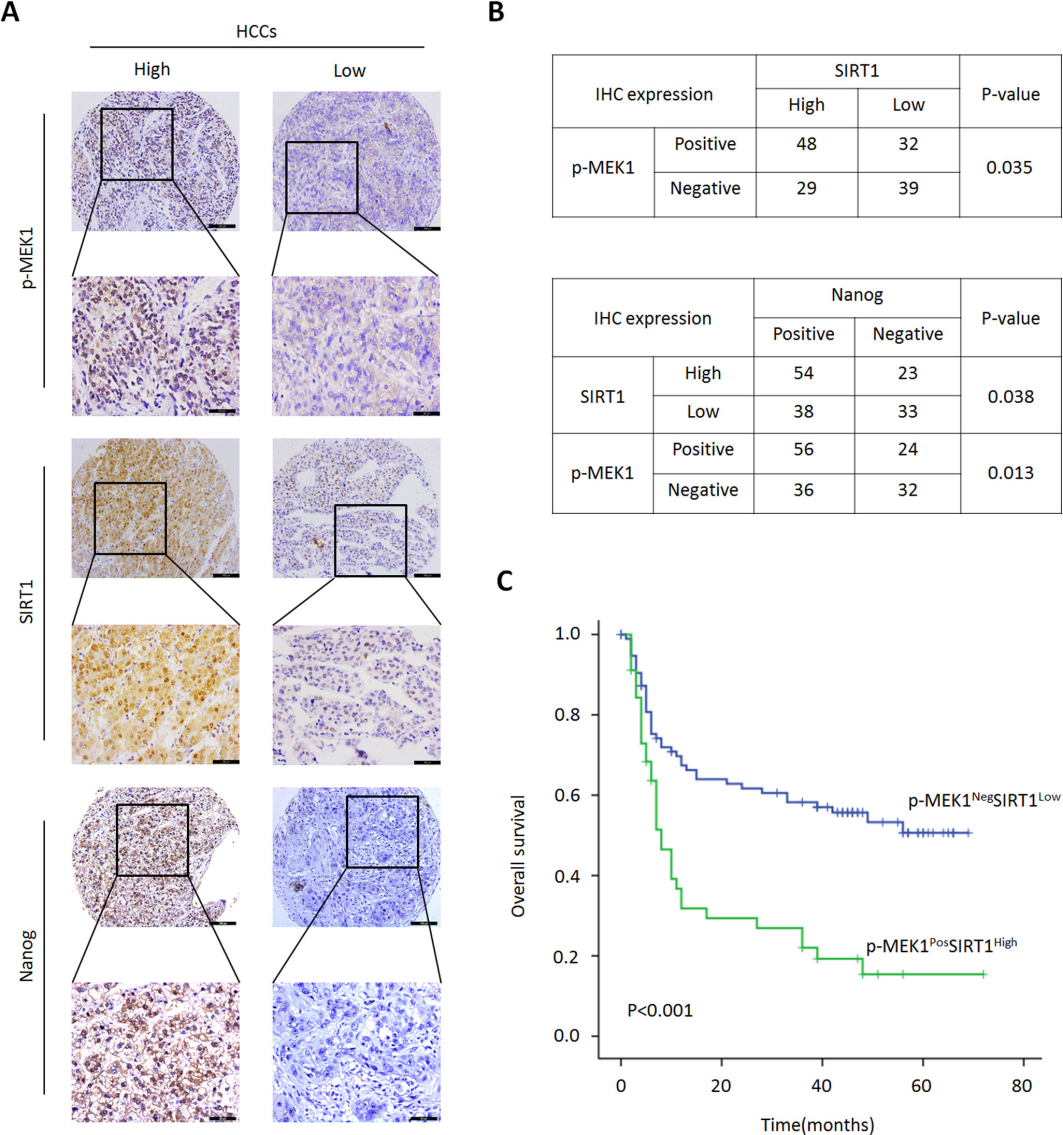
Relationship of p-MEK1/SIRT1 and HCC clinical prognosis (**A**) p-MEK1, SIRT1 and Nanog protein expression were detected by IHC analysis in 148 HCC patients, representative images were shown. (**B**) Analysis correlation between p-MEK1 and SIRT1 expression in 148 HCC patients with Person chi-square test. Correlation analysis of p-MEK1/SIRT1 and Nanog expression in same tissue samples. *P < 0.05 was considered remarkable significant. (**C**) Kaplan-Meier survival analysis was performed according to p-MEK1^Neg^SIRT1^Low^ and p-MEK1^Pos^SIRT1^High^ expression of HCC patients. Survival (*P* < 0.001) of patients who had p-MEK1^Pos^SIRT1^High^ expression was shorter.

Previous study shows that MEK1/MAPK signaling can down-regulate proteins by activating proteasomal degradation [20]. To investigate how MEK1 affects SIRT1 expression and functions on self-renewal, we presumed that MEK1 down-regulated SIRT1 through proteasomal degradation. We discovered that MEK1 could promote SIRT1 expression (Figure 5) and expression of SIRT1 protein was positive correction with MEK1 (Figure 7B). We measured SIRT1 half-life in liver CSCs which treated with cyclohexamide (CHX), an inhibitor of protein translation. When we inhibited proteasome in liver CSCs, high level of SIRT1 expression lasted longer. On the contrary, SIRT1 half-life was shorter after the CSCs treated with U0126, compared with the control (Figure 6A). Knockdown of MEK1 in CSCs led to the same result.

In fact, specific regulation mechanism of SIRT1 protein stability is not well understood. Previous studies implicate that SIRT1 can be degraded by proteasome through ubiquitin-dependent or independent pathways, or through caspase-mediated pathway [21, 22]. We treated liver CSCs with the proteasome inhibitor-MG132 and our results showed that expression of SIRT1 protein was modestly higher (∼2-fold) compared with the untreated (Figure 6B). In addition, the differences in SIRT1 protein expression between control and knockdown of MEK1 were much smaller in MG132-treated cells. Next, we treated cells with MG132 to accumulate ubiquitinated proteins. Consistent with the idea that MEK1 inhibits proteasome degradation of SIRT1, data showed that treatment with U0126 or knockdown of MEK1 expression led to higher SIRT1 ubiquitination level (Figure 6C). These results suggested that the decreased level of SIRT1 mediated by inhibition of MEK1 signaling was dependent on proteasome, which was also responsible for SIRT1 degradation.

### The MEK1/SIRT1 is correlates with poor clinical outcomes in HCC

Finally, we further examined the clinical significance of the MEK1-SIRT1 loop in HCC patients. We used immunohistochemistry to investigate the prognostic significance of the MEK1-SIRT1 profile. The MEK1-SIRT1 loop positive correlation was observed (Pearson Correction = 0.035). The correlation between Nanog expression and MEK1/SIRT1 in tumor tissues was significantly observed in 148 HCC patients (Pearson Correction = 0.013/0.038) (Figure 7A and 7B). Kaplan-Meier's analysis revealed that the MEK1^Pos^ SIRT1^High^ profile was an indicator of the poorest overall survival (*p* < 0.001) for HCC patients (Figure 7C). To gain a better understanding of role of MEK1/SIRT1 in HCC, we gathered and analyzed clinicopathological parameters in the 148 HCC patients. The results were summarized in Table 1. There were no statistical correlation between MEK1/SIRT1 expression and some clinicopathological parameters, such as patient age, gender, AFP level in serum, tumor interstitial hyperplasia, necrosis and recurrence. However, we found the remarkable positive correlation between MEK1/SIRT1 expression and tumor size (*p* = 0.012), vascular invasion (*p* < 0.001), capsular invasion (*p* = 0.048) and clinical tumor stage (*p* < 0.001). Taken together, activation of MEK1 signaling increases SIRT1 stability, which promotes self-renewal and tumorigenicity of liver CSCs resulting in poor prognosis of HCC patients.

**Table 1:** Correction p-MEK1/SIRT1 expression and clinic-pathologic parameters of 87 hepatocellular carcinoma patients

Variable	Total	p-MEK1/SIRT1 (High)	p-MEK1/SIRT1 (Low)	*p*-value
**Age (years)**				
≤ 50	59	30	29	0.541
> 50	24	14	10	
**Gender**				
Female	15	10	5	0.331
Male	72	38	34	
**Tumor stage**				
I	5	1	4	< 0.001***
II	39	16	23	
III	43	31	12	
**Tumor size (cm)**				
< 5	23	7	16	0.012*
≥ 5	59	36	23	
**Serum AFP level (ng/ml)**				
≤ 20	18	9	9	0.721
> 20	62	34	28	
**Tumor recurrence**				
−	45	22	23	0.227
+	42	26	16	
**Necrosis**				
+	38	17	21	0.395
++	26	19	7	
+++	23	12	11	
**Capsular invasion**				
−	27	10	17	0.048*
+	35	22	13	
++	25	16	9	
**Vascular invasion**				
−	25	7	18	< 0.001***
+	62	41	21	
**Interstitial hyperplasia of tumor**				
+	22	13	9	0.969
++	36	18	18	
+++	29	17	12	

Note: (1) *p < 0.05 ***p < 0.001 was considered statistically significant. (2) *P*-values were calculated with the Person chi-square test. (3) Total number < 87 due to missing data.

## DISCUSSION

Recent progresses to understand the biological mechanisms behind the HCC genesis and progression have led to the CSCs, which have been identified by multiple cell surface markers expression [8, 23–28]. These cells have tumor initiating and self-renew properties and promote tumor differentiation, which result in the resistance to chemotherapy and radiotherapy. Our present study provides a fresh insight into the association of the MEK1 in the process of self-renewal of liver CSCs.

It has been reported that MEK1/MAPK signaling was involved in a variety of cancers, such as lung cancer [29], pancreatic cancer [30], prostate cancer [31], melanoma [32] and colon cancer [33]. MEK1/MAPK signaling plays an important role in cancer formation, progression and therapy resistance. Our study provides evidence that inhibition of MEK1 activity significantly decreases liver CSC (Nanog^Pos^) proliferation and self-renewal ability in HCC. To further verify our results, we knocked down MEK1 in CSCs. As expected, self-renewal ability of CSCs is repressed again. It's known that G_1_ cell cycle arrest was induced and apoptosis was increased, when MEK1 was knocked down in bladder cancer [34]. Here, we show that inhibition of MEK1 signaling can significantly retardate liver CSCs in the S/G2 phase, which influences CSCs proliferation and self-renewal.

It is established that the core embryonic stem cell transcriptional circuitry of c-Myc, Sox2, Oct4 and Nanog proteins contribute significantly to the self-renewal functions of CSCs [35]. Supporting this contention, our data showd that inhibition of MEK1 activity or down regulation of MEK1 expression in liver CSCs decrease the expression of C-myc, SOX2, OCT4 and Nanog in CSCs. It has been demonstrated that Ras and B-Raf are known to affect MEK1/MAPK in the control of cell survival [36, 37] and MEK1/MAPK is tightly connected with CLDND1 [38], Foxo3a [39] and p21 [40] in cancers. Thus, MEK1 inhibitors may provide an underlying potential for HCC therapeutic intervention, either alone or in combination with other drugs.

SIRT1 is a class III histone deacetylase which located both in nuclear and cytoplasm [41]. Recently reports indicate that SIRT1 participates in self-renewal and differentiation of both human hematopoietic stem cells and mouse embryonic stem cells [42–44]. Thus, we hypothesizes that MEK1 regulates sirtuins, SIRT1 most possibly, to influence tumorigenesis and self-renewal of liver CSCs. Our data affirm this idea that MEK1 inhibition or knockdown can markedly repress SIRT1 expression. Additional research demonstrates that overexpression of exogenous SIRT1 can reverse the decreased self-renewal and tumorigenesis of liver CSCs induced by MEK1 inhibition. Meanwhile, overexpression of SIRT1 enhances non-CSCs ability of self-renewal, and inhibition MEK1 activity terminates this promotion. Our conclusion just offers a new site to regulate SIRT1.

More and more evidence show that ubiquitin-proteasome pathway is associated with cancer [45, 46]. People find that TGF-β treatment lead to protein degradation of PTHrP through the ubiquitin-proteasome-dependent pathway [47]. Research shows that inhibition of Raf-MEK-ERK pathway by Cyclic AMP signaling can promote ubiquitin-proteasomal degradation to reduce SIRT6 expression in non-small cell lung cancer cells [48]. With the effect of MG132 and CHX, we discover that MEK1 promotes SIRT1 expression through inhibiting proteasome-mediated degradation. Additionally we confirm that knockdown or inhibition MEK1 can improve SIRT1 ubiquitin state. In summary, MEK1 promotes SIRT1 ubiquitination to suppress protein degradation.

All in all, our studies show a novel mechanism that MEK1 inactivation inhibits HCC tumorigenesis *in vitro* and *vivo* by promoting SIRT1 ubiquitination which result in SIRT1 protein degradation. Considering the fact that poly (ethylene glycol)-b-poly (d, l-lactide) (PEG-PLA) nanoparticles encapsulation enhances the cell uptake of U0126 in HCC CSCs [49]. It is promising that MEK1 may represent potential therapeutic targets for HCC in the bright future.

## MATERIALS AND METHODS

### Cell culture and lentiviral transductions

The human HCC cell lines (PLC/PRF/5 and Huh-7) and 293T were cultured and conserved by Southwest Cancer Center, Southwest Hospital, Third Military Medical University from 2009 and have been used in previous study [8]. The cells were grown in DMEM medium (Invitrogen) supplemented with 10% fetal bovine serum (FBS) (Gibco) at 37°C in a humidified atmosphere containing 5% CO_2_.

Lentiviruses were prepared in 293T packaging cells via transfection with a four-plasmid system. Transfections were performed in 100 mm plates. Packaging cells were seeded at 3.5 × 10^5^ cells per plate in DMEM/10% FBS 24 hr before transfection and grown at 37°C/5% CO_2_. DNA for transfection was prepared by mixing 15 μg of shRNA/gene-encoded plasmid, 4 μg pRRE, 3 μg pREV and 6 μg pMD2.G, which were mixed with 50 μl CaCl_2_ (1.25 M) and 500 μl of 2 × HBS in a final volume of 1 mL and allowed to complex for 20 min at room temperature before addition to the packaging cells. Cells were incubated overnight and the transfection reagent was subsequently removed and exchanged for DMEM/10% FBS. Lentiviral supernatants from 48 and 72 hrs were pooled, filtered through a 0.45 μm filter and lentivirus was frozen at −80°C until used to infect cells.

For shRNA or gene-encoded lentivirus mediated knockdown or overexpression experiments, cells were infected with the same virus MOI. After an overnight incubation and the medium was refreshed the following day and polybrene was added 72 hr post-infection at a final concentration of 2 μg/mL. Protein expression was analyzed by immunoblotting after 72 hr of selection.

### Clinical specimens

Tumorous liver tissue samples and the corresponding adjacent nontumoral liver tissue samples were obtained from 148 HCC patients who underwent curative surgery at the Institute of Hepatobiliary Surgery, Southwest Hospital, Third Military Medical University. Informed consent was obtained from each patient that was recruited.

### Animal

All NOD/SCID mice were used in this research and obtained from the Third Military Medical University and were maintained at pathogen-free conditions. All procedures were done according to protocols approved by the Institutional Review Board of the Southwest Hospital, Third Military Medical University and conformed to the NIH guidelines on the ethical use of animals. Mice were 4–5 weeks of at age of injections. Tumors were dissected at the end of the experiments and weighed.

### Knockdown using shRNAs

shRNAs were designed using iRNAi software (Mekentosj) to meet the following criteria: 19 nucleotides in length, 45%–55% G/C content, higher free energy in the 30 antisense region compared to the 50 antisense region. To avoid off-target effects, a BLAST (NIH) homology search was performed on each shRNA sequence candidate. A minimum of two or three shRNAs were designed for each protein to be knocked down. shRNAs were cloned into the lentiviral plasmid to be expressed. The efficacy of each shRNA was assessed by western blotting of endogenous protein that had been infected with the viruses upon plating and were cultured for 3 days. The shRNAs with the strongest knockdown efficiency were selected for further experiment so long as they knocked down greater than 75% of the protein. Oligonucleotide sequence of shRNAs as following:

Scrambled shRNA: 5′-CGTACGCGGAATACTTCGA-3′

MEK1 shRNA#1: 5′-CTCTGGATCAAGTCCTGAACTC-3′

MEK1 shRNA#2: 5′GGACTCATTACTCTGTGCACTC-3′.′

### Flow cytometry

Cells were prepared according to standard protocols. Samples were sorted and analyzed using BD FACS Aria II (BD Biosciences).

### Inhibitor(s) treatment *in vitro*

Different milligrams of inhibitor(s) were dissolved in dimethyl sulfoxide (DMSO) or enhanol. Cells were plated in six-well plates. When the cells reached 60% confluency, they were treated with different doses inhibitor (s) or different time at the same dose of inhibitor (s), and cells were collected after treatment for protein extraction and analyzed by immunoblotting.

### Tumor information

Generally, 1 × 10^6^ cells or at the range of cells from 100 to 10,000 cells mixed with matrigel were injected subcutaneously into the NOD/SCID mice. Tumors grew for approximately 4 weeks or longer time until they reached an appropriated size (150–200 mm^3^). Tumor size was measured with a digital caliper and calculated with the help of the following formula: length × (width)^2^ × π/6.

### Immunoprecipitation

Cells were collected and lysed in lysis buffer (Thermo) supplemented with protease inhibitors, incubated on ice for 30 min, and cleared by centrifugation at 13,500 rpm at 4°C for 15 min. Total protein lysate (600 μg) was subjected to immunoprecititation with the agarose-immobilized antibody (anti-SIRT1 antibody) for overnight at 4°C. After incubation, protein immunocomplexes were washed 4 times with 800 μl of wash buffer (Thermo). Protein complexes were analyzed by immunoblotting using 8%–15% SDS-PAGE gel.

### Statistical analysis

Data are presented as mean ± SEM. All statistical analyses were conducted using GraphPad Prism Software Version 6.0 (GraphPad Software Inc, La Jolla, CA). Statistical significance is represented as *p < 0.05, **p < 0.01 or ***p < 0.001.

### Immunofluorescence

For immunofluorescence, cells were cultured in 24-well on glass cover-slips, and washed three times with PBS before fixed in 4% paraformaldehyde and permeabilized with 0.5% Triton X-100. Cells were blocked with 10% FBS (Gibco) in PBS for 30 min at room temperature (RT). Wash steps and ki67 antibody incubation steps. Secondary antibodies were donkey anti-rabbit IgG-Alexa Fluor 647 was purchased from Invitrogen. Cells were further washed in PBS and mounted with vectashield mounting medium containing 40, 6-diamidino-2-phenylindole (DAPI) for counterstaining nuclei. Cells were analyzed by using fluorescence microscopy.

### Immunoblotting

Cells were collected and lysed in NP40 lysis buffer (Thermo) supplemented with protease inhibitors cocktail and phosphatase inhibitors, incubated on ice for 30 min and cleared by centrifugation at 4°C for 15 min. Protein lysates were equalized and analyzed by western blotting using 8%–15% SDS-PAGE gel and proteins were liquid transferred to nitrocellulose membrane in transfer buffer (20% methanol, 25 mM Tris, 192 mM Glycine, 0.037% SDS) during 120 min at 100 V. Membranes were blocked with 5% nonfat drymilk and PBS 0.1% tween-20 for 2 hr at 37°C with gentle shaking, and then incubated overnight at 4°C with specific primary antibodies as described in Antibodies and incubated 2 hr at RT in PBS 0.1% tween-20. Either anti-rabbit IgG or anti-mouse IgG horseradish peroxidase-conjugated secondary antibodies incubated 1 hr at RT in PBS-5% milk-0.1% tween-20, and immunocomplexes were visualized by using SuperSignal West Femto Chemiluminescent Substrate (Pierce). For quantification, signals were densitometrically normalized to GAPDH by GeneTools image analysis program (SynGene).

### Colony formation assays

Briefly, 1 × 10^4^ cells were seeded in 10-cm tissue culture plates or 200 cells were seeded in 24 well plates. The cells were cultured in the DMEM medium supplemented with 10% of FBS in the absence or presence of different concentrations of MEK1 inhibitors for 14 days. The colonies were fixed with 4% formaldehyde and stained with 0.1% crystal violet (Sigma–Aldrich). Numbers of clones were counted.

### Sphere formation analysis

A total of 200 the sorted Nanog^Pos^ or Nanog^Neg^ cells were plated into Costar^®^ Ultra Low Cluster 24-well plates (Corning). The cells were cultured in the DMEM/F12 medium (Sigma) supplemented with B27 (Gibco), antibiotics, 20 ng/mL EGF, 20 ng/mL bFGF (Peprotech) and 10 ng/mL HGF (Peprotech) in the absence or presence of different concentrations of MEK1 inhibitors, and 1% methyl cellulose was added to prevent cell aggregation. Cells were incubated at 37°C for 7 days and numbers of spheres were counted.

### Analysis of cell-cycle distribution

Cell-cycle distribution was determined by fluorescence-activated cell sorting (FACS) analysis, as previously described Cells were stained with propridium iodide (PI; Sigma-Aldrich). Flow cytometry was carried out by a FACS Calibur flow cytometer (BD Biosciences, San Jose, CA). Data acquisition and analysis were done with CellQuest (BD Biosciences).

### Proliferation assay

The cells were seeded in a 96-well plate (Thermo) at 1000-3000 cells per well, Cell viability was measured by CCK-8 proliferation assay and was performed according to the manufacturer's instructions (Dojindo Laboratory, CK04) for 6 days.

### Immunohistochemical (IHC) staining

IHC staining was performed on clinical samples through the streptavidin biotin peroxidase complex method. The antigen retrieval procedure was heated in a pressure cooker with 10 mmol/L EDTA (pH 8.0) of Dako antigen retrieval solution. Then, the samples were stained with following primary antibodies, rabbit anti-human SIRT1 (Santa Cruz), rabbit anti-human Nanog (abcam) or rabbit anti-human phospho-MEK1 (Cell Signaling). Samples were subsequently developed using EnVision method with a DAKO kits (Dako REALTM EnVisionTM). Scoring for IHC staining was performed by two independent pathologists. We quantitatively scored the tissue sections according to the percentage of positive cells and the staining intensity. We assigned the following proportion scores: 0 (0–1%), 1 (1–25%), 2 (26–50%), 3 (51–75%) and 4 (76–100%). We rate the intensity of staining on a scale of 0 to 3: 0 for negative; 1 for weak; 2 for moderate; and 3 for strong. The positivity and intensity scores were combined to protein expression score (overall score range, 0–12). Scores were compared with overall survival, defined as the time from date of surgery to death or last known date of follow-up.

### Chemical and reagents

Inhibitors with the follows: U0126 (662005) were purchased from Merck. DAPT (D5942) and Cycloheximide (N11534) were purchased from Sigma. MG-132 (ab141003) was purchased from abcam. PD98059 (9900) were purchased from Cell Signal Technology.

### Antibodies

SOX2 (2748), MEK1 (12671), phospho-MEK1 (2338), Ki-67 (8D5) (9449) and GAPDH (2118L) antibodies were purchased from Cell Signaling Technology. SIRT1 (H-300) (sc-15404), c-Myc (9E10) (sc-40) and Ub (sc-8017) antibodies were purchase from Santa Cruz. GFP (ab290), OCT4 (ab19857) and Nanog (ab109250) antibodies were purchased from abcam. ERK1/2 (05-1152) and phospho- ERK1/2 (05-797R) were purchased from Millpore. Alexa Flour 647 (A31571) antibody was purchased from life technologies.

### Quantifications

Quantifications of immunobloting were achieved using the NIH Image J software (http://rsb.info.nih.gov/ij/).

## SUPPLEMENTARY MATERIALS FIGURES AND TABLE


