# Supplementary material for: MEK1 signaling promotes self-renewal and tumorigenicity of liver cancer stem cells via maintaining SIRT1 protein stabilization
Source: Oncotarget. 2016 Mar 7;7(15):20597–611. doi: 10.18632/oncotarget.7972 (PMC4991478; doi:10.18632/oncotarget.7972)
Supplement: Supplementary file 1 [file oncotarget-07-20597-s001.pdf]

MEK1 signaling promotes self-renewal and tumorigenicity of liver cancer stem cells via maintaining SIRT1 protein stabilization

Supplementary Materials

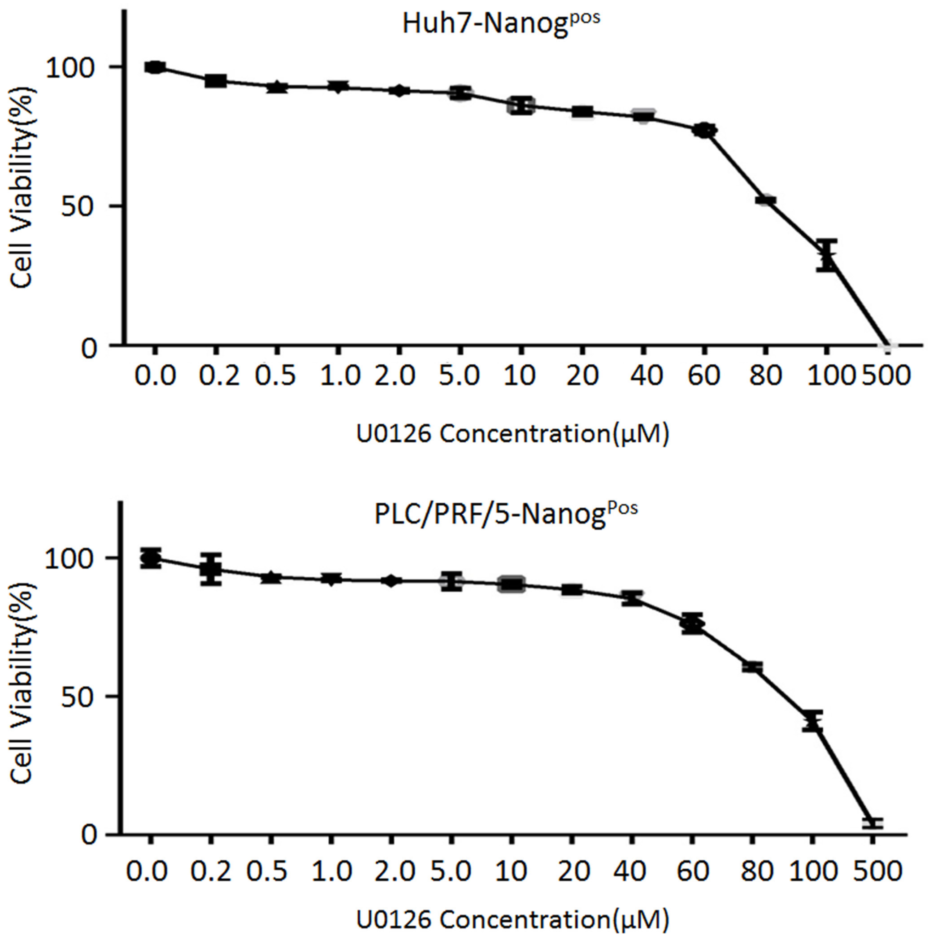

Supplementary Figure S1: Toxicity test of U0126 to liver CSCs. CSCs (Huh7- and PLC/PRF/5- Nanog^{Pos} cells) were treated with indicated concentration of U0126 for 24 hours, before being analyzed with the CCK8 assay.

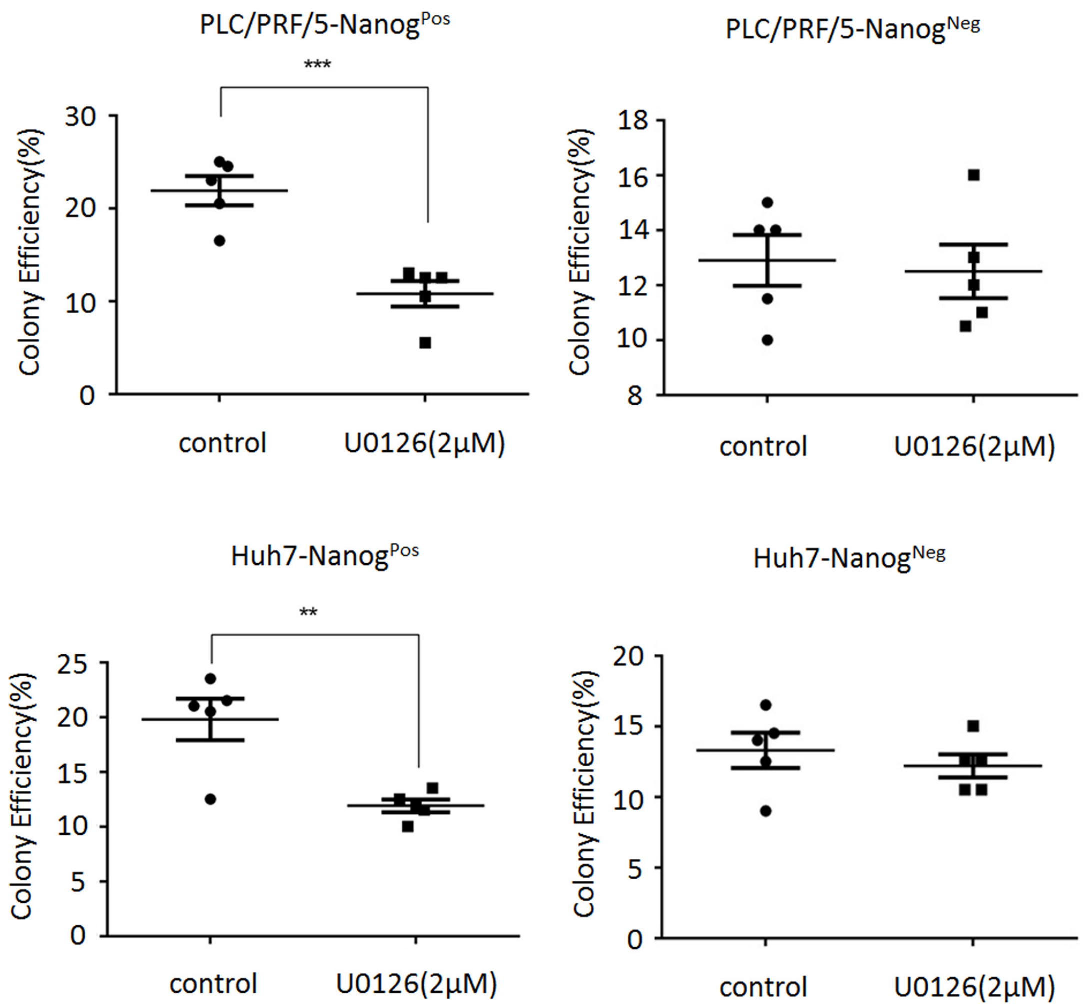

Supplementary Figure S2: U0126 inhibited CSCs and non-CSCs discrepantly in Colony capacity. CSCs and non-CSCs were treated with 2 μM U0126 for 14 days and stained with crystal violet.

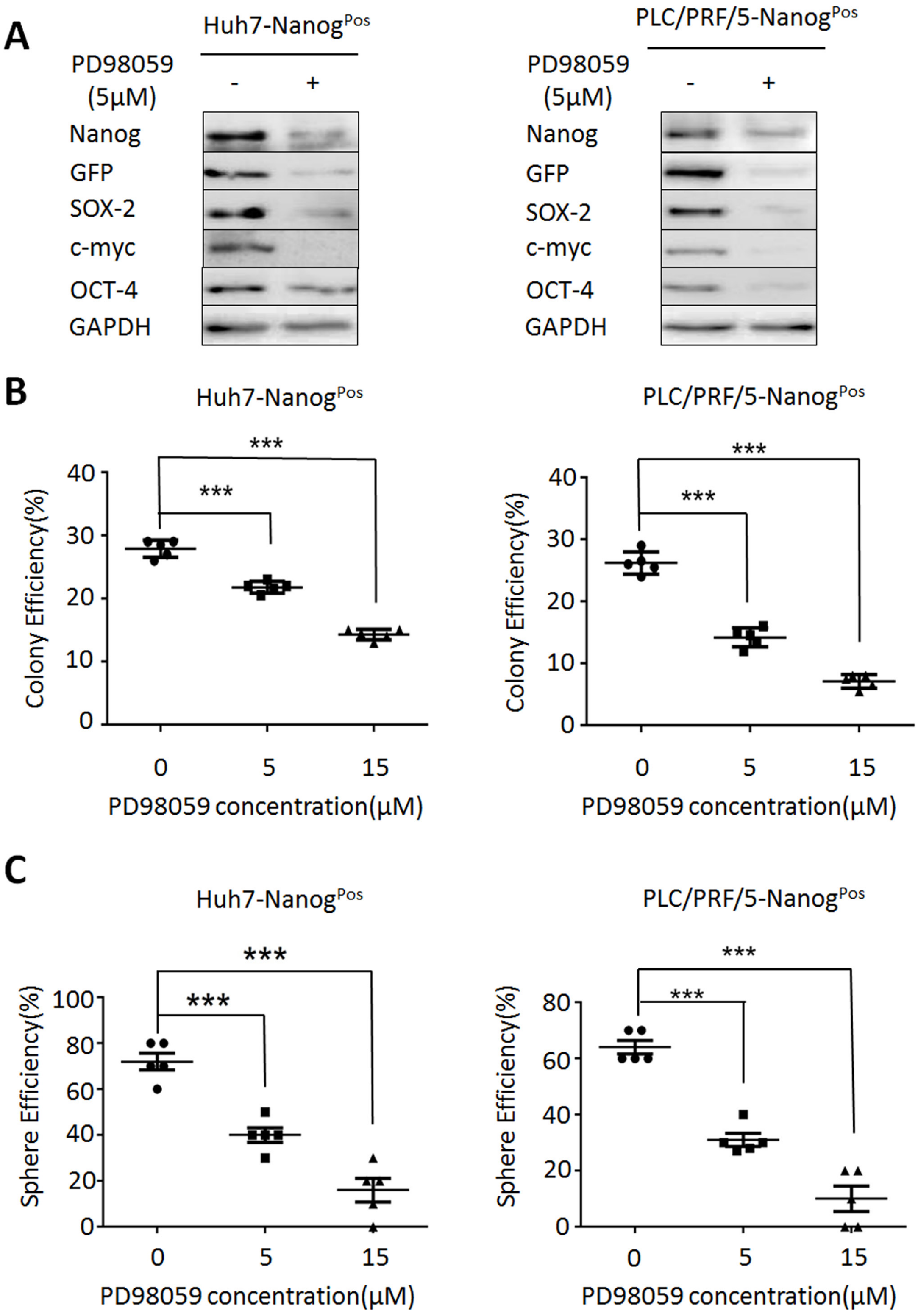

Supplementary Figure S3: PD98059 significantly inhibited CSCs self-renewal and tumorigenesis. (**A**) Proteins were obtained after 5 µM PD98059 treatment of CSCs for 48 hours. Western blots analysis of Nanog, GFP, SOX2, OCT4 and c-myc expression level compared with negative control. (**B**) CSCs were co-cultured with indicate dose of PD98059 for 14 days before stained with crystal violet and counted. (**C**) CSCs were treated with 5 µM or 15 µM PD98059 in non-adhesion system for 7 days before counted.

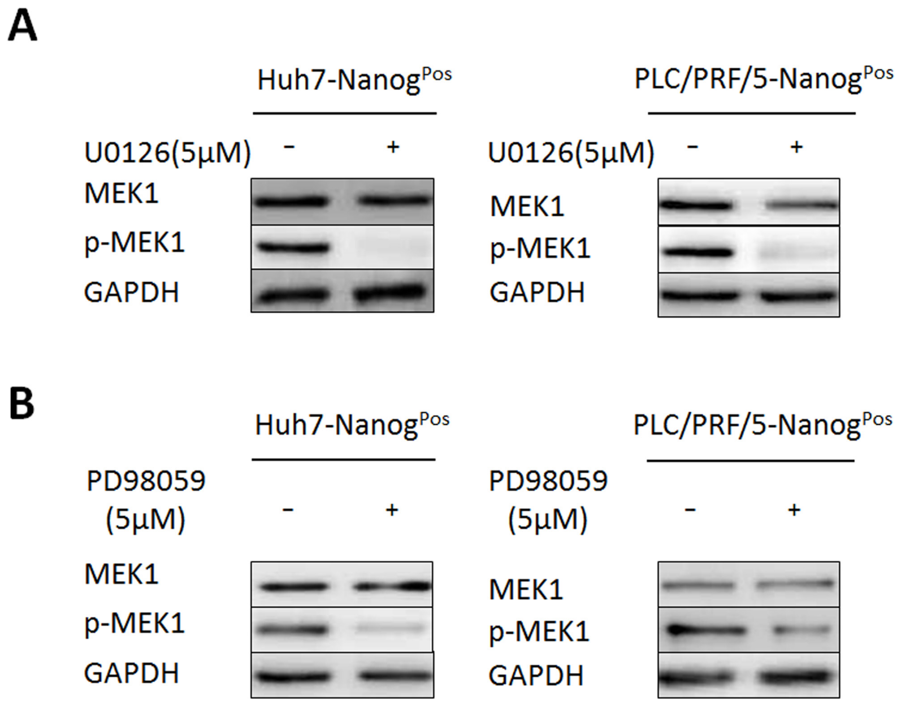

Supplementary Figure S4: MEK1 inhibitor inhibited phosphorylated-MEK1 expression. (**A–B**) CSCs (Huh7- and PLC/PRF/5-Nanog^{Pos} cells) were treated with 5 μM U0126 or 5 μM PD98059 for 48 hours. Cells were harvested and analysis by western blots about the expression of MEK1 and phosphorylated-MEK1.

Detail of Antibody

Name	Company	Catalog Number	Dilutions
SOX2	Cell Signaling Technology	2748	1:1000 for WB
MEK1	Cell Signaling Technology	12671	1:500 for WB
phospho-MEK1	Cell Signaling Technology	2338	1:500 for WB, 1:50 for IHC
GAPDH	Cell Signaling Technology	2118L	1:1000 for WB
Ki-67	Cell Signaling Technology	9449	1:800 for IF
SIRT1 (H-300)	Santa Cruz	sc-15404	1:500 for WB,1:200 for IP,1: 50 for IHC
c-Myc (9E10)	Santa Cruz	sc-40	1:500 for WB
Ub	Santa Cruz	sc-8017	1:500 for WB, 1:300 for IP
GFP	abcam	ab290	1:1000 for WB
OCT4	abcam	ab19857	1:1000 for WB
Nanog	abcam	ab109250	1:1000 for WB, 1:100 for IHC
ERK1/2	Millpore	05-1152	1:1000 for WB
Phospho-ERK1/2	Millpore	05-797R	1:1000 for WB
Alexa Flour 647	life technologies	A31571	1:1000 for IF
